# Modeling the ascorbate-glutathione cycle in chloroplasts under light/dark conditions

**DOI:** 10.1186/s12918-015-0239-y

**Published:** 2016-01-22

**Authors:** Edelmira Valero, Hermenegilda Macià, Ildefonso M. De la Fuente, José-Antonio Hernández, María-Isabel González-Sánchez, Francisco García-Carmona

**Affiliations:** Department of Physical Chemistry, School of Industrial Engineering, University of Castilla-La Mancha, Campus Universitario, s/n, Albacete, E-02071 Spain; Department of Mathematics, School of Computer Science, University of Castilla-La Mancha, Campus Universitario, s/n, Albacete, E-02071 Spain; Institute of Parasitology and Biomedicine “López-Neyra”, CSIC, Granada, Spain; Department of Mathematics, University of the Basque Country, UPV/EHU, Leioa, Spain; Department of Plant Breeding, CEBAS, CSIC, Group Fruit Trees Biotechnology, Murcia, Spain; Department of Biochemistry and Molecular Biology A, Biology Faculty, University of Murcia, Murcia, E-30100 Spain

**Keywords:** Light/dark cycles, Ascorbate-glutathione cycle, Computer simulation, Oxidative stress, Reactive oxygen species, Chloroplast

## Abstract

**Background:**

Light/dark cycles are probably the most important environmental signals that regulate plant development. Light is essential for photosynthesis, but an excess, in combination with the unavoidable presence of atmospheric oxygen inside the chloroplast, leads to excessive reactive oxygen species production. Among the defense mechanisms that activate plants to cope with environmental stress situations, it is worth noting the ascorbate-glutathione cycle, a complex metabolic pathway in which a variety of photochemical, chemical and enzymatic steps are involved.

**Results:**

We herein studied the dynamic behavior of this pathway under light/dark conditions and for several consecutive days. For this purpose, a mathematical model was developed including a variable electron source with a rate law proportional to the intensity of solar irradiance during the photoperiod, and which is continuously turned off at night and on again the next day. The model is defined by a nonlinear system of ordinary differential equations with an on/off time-dependent input, including a parameter to simulate the fact that the photoperiod length is not constant throughout the year, and which takes into account the particular experimental kinetics of each enzyme involved in the pathway. Unlike previous models, which have only provided steady-state solutions, the present model is able to simulate diurnal fluctuations in the metabolite concentrations, fluxes and enzymatic rates involved in the network.

**Conclusions:**

The obtained results are broadly consistent with experimental observations and highlight the key role played by ascorbate recycling for plants to adapt to their surrounding environment. This approach provides a new strategy to in vivo studies to analyze plant defense mechanisms against oxidative stress induced by external changes, which can also be extrapolated to other complex metabolic pathways to constitute a useful tool to the scientific community in general.

**Electronic supplementary material:**

The online version of this article (doi:10.1186/s12918-015-0239-y) contains supplementary material, which is available to authorized users.

## Background

The Earth takes approximately 24 h to make one complete turn around its own axis, which gives rise to the succession of day and night. In the daytime, the amount of sunlight that reaches the surface of our planet is not constant, but varies according to different factors such as time of day, season, altitude, latitude and atmospheric composition. The length of the light and darkness periods is not the same, and neither is constant throughout the year. Living organisms have evolved to coordinate their activities with light/dark cycles, which greatly influence many aspects of their metabolism, physiology, and even their behavior.

In plants, chloroplasts are the organelles that capture sunlight energy and store it as chemical energy to be used in photosynthesis. Plants have evolved different mechanisms to cope with natural fluctuations in light intensity to not only be able to harvest light optimally, but to also protect themselves from excess light [1]. When the light absorbed/light used for CO_2_ fixation ratio is above 1, the generation of reactive oxygen species (ROS) is greatly accelerated, which will lead to the inhibition of photosynthetic machinery. Under such conditions, plants can activate different protective mechanisms to dissipate excess photon energy, including photorespiration, down-regulation of photosystem II (PSII) through proton gradient generation across thylakoid membranes, cyclic electron flow around the PSI or the water-water cycle [2, 3]. In addition, plants can also dissipate excess excitation energy in PSII antenna as heat through nonphotochemical processes, which involves the xanthophyll cycle using ascorbate (ASC) as the reducing agent [4].

**Fig. 1:**
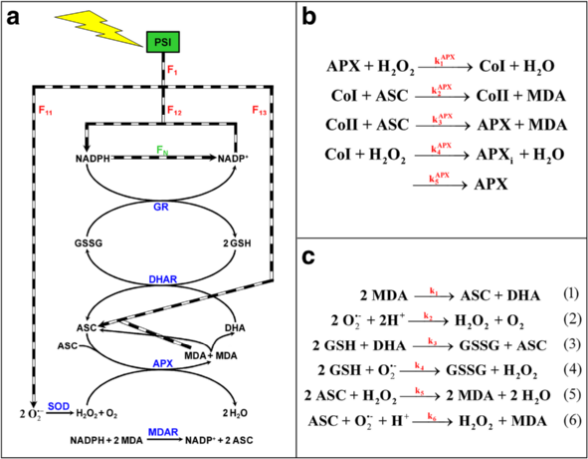
Metabolic network designed to simulate the ASC-GSH pathway in chloroplasts under light/dark conditions. **a** The light-dependent processes and enzymatic steps involved in the model; black and white checkered arrows indicate the steps which are light-dependent, so they are switched off under dark conditions. **b** the APX catalytic mechanism introduced into the network, including the inactivation of the enzyme by the reaction of CoI with H_2_O_2_ and continuous APX input into the system by *de novo* synthesis of the protein. **c** The spontaneous reactions included in the model; *k*_*j*_ values (*j* = 1–6) were considered apparent bimolecular rate constants

ROS are generally toxic and can cause damage to different biomolecules. So their fast removal is crucial for plant survival. For this purpose, plants have developed a set of antioxidants such as glutathione (GSH) and ASC, as well as antioxidant enzymes such as superoxide dismutase (SOD) and ascorbate peroxidase (APX). GSH, ASC and APX work in the so-called ASC-GSH cycle together with other enzymes, which include glutathione reductase (GR), dehydroascorbate reductase (DHAR) and monodehydroascorbate reductase (MDAR) [2, 3]. The metabolic pathway consists in two coupled substrate cycles [5] (Fig. 1a) to achieve high sensitivity and amplification of the response [6, 7]. The dynamic behavior of this pathway has been previously analyzed by computer simulation [5, 8], which makes it possible to calculate concentrations of the species and fluxes of the steps involved in the proposed mathematical models in both unstressed and stressed chloroplasts. However, the reaction schemes used in these reports included a constant electron source, so they did not address the effect of the variations of solar irradiance intensity and were only valid under fixed light conditions. Nevertheless, it has been shown that the antioxidants concentrations and enzymatic activities involved in the detoxifying pathway are subject to diurnal fluctuations, with significant changes relating to changes in light intensity over the 24 h period [9–11]. Furthermore, it has also been reported that ROS responsive genes are not under circadian control, but their transcription is induced upon stress, and that the activity of many ROS-scavenging enzymes depends on the redox state of the plant cell [12]. Understanding all the simultaneous metabolic responses against the different light/dark conditions is currently one of the major challenges in plant research to improve crop productivity under a changing global climate.

In the present paper, our goal was to develop a mathematical model based on ordinary differential equations (ODEs) that would allow us to simulate the dynamic behavior of the species involved in the ASC-GSH pathway for several consecutive days when an electron flux is introduced into the model by means of a rate law proportional to the intensity of solar irradiance, which is switched off under dark conditions. Light is one of the most important environmental factors to affect plant growth. Hence it should be considered in mathematical models to gain insights into the dynamics of plant physiological processes. The model developed herein represents an open system that allows the analysis, from a quantitative viewpoint, of the effect of such an environmentally relevant factor like sunlight on the temporal dynamics of the metabolites and fluxes involved in a metabolic network, particularly the H_2_O_2_-detoxifying pathway in chloroplasts. This tool is straightforwardly extendible to other light-dependent biological processes that occur in plants and other living organisms.

### Model

The metabolic pathway under study is shown in Fig. 1. First of all, the model includes an electron source, PSI, which is active during the photoperiod, but is switched off at night. Therefore, the electron flow from PSI, *F*_*1*_, is an input into the system which has been included as an on/off switch. It is a time-dependent variable that has been divided into three competitive routes, which obviously do not work under dark conditions. They are the following:

- photoreduction of O_2_ to O_2_^· −^ (one-electron), whose flux is *F*_*11*_
- photoreduction of NADP^+^ to NADPH (two-electrons), whose flux is *F*_*12*_
- photoreduction of monodehydroascorbate (MDA) to ASC (one-electron), whose flux is *F*_*13*_,

where *F*_*1*_ *= F*_*11*_ *+ F*_*12*_ *+ F*_*13*_. To express this fact mathematically, three distribution coefficients were defined, *c*_*11*_, *c*_*12*_ and *c*_*13*_, respectively, whose sum equals unity; i.e. *c*_*11*_ + *c*_*12*_ + *c*_*13*_ = 1. These coefficients are the following:

7 $$ {c}_{11}=\frac{k_{11}}{k_{11}+2{k}_{12}\left[NAD{P}^{+}\right]+{k}_{13}\left[MDA\right]} $$

8 $$ {c}_{12}=\frac{2{k}_{12}\left[NAD{P}^{+}\right]}{k_{11}+2{k}_{12}\left[NAD{P}^{+}\right]+{k}_{13}\left[MDA\right]} $$

9 $$ {c}_{13}=\frac{k_{13}\left[MDA\right]}{k_{11}+2{k}_{12}\left[NAD{P}^{+}\right]+{k}_{13}\left[MDA\right]} $$

In this way, the expression for each electron flow is as follows:

10 $$ {F}_j={c}_j{F}_1\kern0.5em \left(j = 11,12,13\right) $$

Therefore the distribution of the electron flow among the three branches is not always the same, but varies throughout the photoperiod, and could even differ from day to day, depending on the redox conditions of the chloroplast (particularly on the MDA and NADP^+^ concentrations). This confers the model much flexibility. The O_2_ and CO_2_ concentrations were considered constant in the model.

To establish the most appropriate mathematical function that describes variation in *F*_*1*_ with time during the photoperiod, the following assumptions were made: *F*_*1*_ is zero at the beginning and the end of the photoperiod, it shows its maximum at noon, and it follows a rate law proportional to the solar irradiance intensity. So experimental data of average daily global solar irradiance for different months and cities were taken from [13] and were statistically fitted to different kinds of mathematical equations. The next sinusoidal function was chosen because it provided a good enough fit to experimental data (an example of the goodness-of-fit of real average daily global solar irradiance data to Eq. (11) is illustrated in Additional file 1, for an inland city of Spain):

11 $$ y={y}_0+{a}_i \sin \left(\frac{2\pi t}{b}+c\right) $$

Furthermore, the sinusoidal function offers the additional advantage of a clearly established physical meaning of the parameters (*y*_*0*_ is a parameter to displace the *sin* function in the ordinate axis to avoid negative values, *a*_*i*_ is the amplitude, *b* is the inverse of frequency and *c* is the phase). For greater comprehensibility, we used the condition *y*_*0*_ *= a*_*i*_ (*a*_*i*_ > 0), so that the maximum value of *F*_*1*_ was *2a*_*i*_ and the minimum value was 0. Therefore in our model, *2a*_*i*_ was the maximum value of *F*_*1*_ at noon, *b* was the duration of the photoperiod in hours (if Δ = 0 and/or *i* = 0, see below) and *c* = 3π/2. A day counter, *i*, was included in the model to write the equations in a more simplified manner. Its expression is the following:

12 $$ i= floor\left( Time/24\right) $$

The *a*_*i*_ value can be the same for every day. However, the intensity of solar irradiance is not the same every day; so *F*_*1*_ varies from day to day. For this reason, a different value of *a*_*i*_ was included in the model for each day, which was randomly obtained by a discrete uniform distribution in the COPASI software [14]:

13 $$ {a}_i= uniform\left( \min, \max \right) $$

We must also take into account that the length of the photoperiod is not the same every day because it depends on the season of the year under study. This fact was included in the model by the parameter Δ, whose value can be positive or negative (or null) depending on whether the length of the photoperiod became longer or shorter (or constant) from one solstice to the other. It is important to note here that full day duration (light and dark conditions) was considered to be 24 h, where (*b + i*Δ) is the length of the photoperiod and 24 – (*b + i*Δ) the length of night. Simulation started and ended each photoperiod under dark conditions, i.e. *F*_*1*_(24*i*) = 0, *F*_*1*_(24*i* + *b* + *i*∆) = 0 and *2a*_*i*_ > *F*_*1*_(*Time) >* 0 if 24*i < Time <* 24*i* + *b* + *i*∆. After bearing all this in mind, *F*_*1*_ was defined as follows:

One important feature of the pathway under study is the existence of three moiety-conserved cycles (metabolic structures interconverting different forms of a chemical moiety, while the sum of these forms remains constant [15–17]), which are the sums (NADPH + NADP^+^), (GSH + 2GSSG) and (ASC + DHA + MDA) (Fig. 1a). NADPH is photoproduced from NADP^+^ by light-dependent reactions (*F*_*12*_); the antioxidant power of NADPH is then used to regenerate GSH and ASC by the GR- and MDAR-catalyzed reactions through the reduction of GSSG and MDA, respectively; an additional step of NADPH consumption by the Calvin-Benson cycle (and other electron-consuming reactions) with flux *F*_*N*_ was also added to the model, as described in [5]. The equation that describes *F*_*N*_ is the following:

15 $$ {F}_N={k}_N\left[ NADPH\right] $$

where:

16 $$ {k}_N={k}_{N,cte}{F}_{12} $$

and *k*_*N,cte*_ is the apparent rate constant for the flow *F*_*N*_.

GSH and GSSG are interconverted each other by the action of the enzymes GR and DHAR. MDA radicals are produced following ASC oxidation by the APX-catalyzed H_2_O_2_ reduction, and spontaneously undergo disproportionation into dehydroascorbate (DHA) and ASC. DHA is recycled into ASC by DHAR. ASC is also recovered following MDA reduction by both *F*_*13*_ and MDAR. Given that MDA has been shown to be mainly photoreduced via ferredoxin (*F*_*13*_), but not via NAD(P)H with MDAR, at least in the thylakoidal scavenging system [2], MDAR was only included into the model when indicated in the text, and NADPH was employed as the electron donor.

The four enzymes involved in the metabolic pathway, GR, DHAR, MDAR and APX, were considered active 24 h a day whenever their respective substrates were available. SOD was also included under the same conditions, but given its high catalytic efficiency [18], it was evident that it would work only under light conditions when superoxide radicals are generated in the model (*F*_*11*_). Each enzyme was introduced into the model after taking into account its particular mechanism of action based on previously reported experimental data (for further details see [5]), namely for GR [19] and MDAR [20], the ping-pong mechanism:

17 $$ {V}_{GR}=\frac{k_{cat}^{GR}{\left[GR\right]}_0\left[ NADPH\right]\left[ GSSG\right]}{K_{m,GR}^{NADPH}\left[ GSSG\right]+{K}_{m,GR}^{GSSG}\left[ NADPH\right]+\left[ NADPH\right]\left[ GSSG\right]} $$

18 $$ {V}_{MDAR}=\frac{k_{cat}^{MDAR}{\left[ MDAR\right]}_0\left[ NADPH\right]\left[MDA\right]}{K_{m, MDAR}^{NADPH}\left[MDA\right]+{K}_{m, MDAR}^{MDA}\left[ NADPH\right]+\left[ NADPH\right]\left[MDA\right]} $$

For DHAR, a bi uni uni uni ping-pong mechanism [21]:

19 $$ {V}_{DHAR}=\frac{k_{cat}^{DHAR}{\left[ DHAR\right]}_0\left[DHA\right]\left[GSH\right]}{K_i^{DHA}{K}_{m, DHAR}^{GSH1}+{K}_{m, DHAR}^{DHA}\left[GSH\right]+\left({K}_{m, DHAR}^{GSH1}+{K}_{m, DHAR}^{GSH2}\right)\left[DHA\right]+\left[DHA\right]\left[GSH\right]} $$

For SOD, after taking into account its remarkably high activity as an O_2_^· −^ scavenger, and the fact that the O_2_^· −^ levels inside chloroplasts are well below *K*_*m*_ (350 μM) [22], first-order kinetics were assumed:

20 $$ {V}_{SOD}={k}^{SOD}{\left[ SOD\right]}_0\left[{O}_2^{\cdot -}\right] $$

In the particular case of APX, a more detailed mechanism was introduced with the next steps (Fig. 1b): 1) reaction of native enzyme with H_2_O_2_ to yield Compound I of APX (CoI); 2) oxidation of ASC to MDA by CoI; 3) oxidation of ASC to MDA by Compound II of APX (CoII); 4) H_2_O_2_-induced inactivation of the enzyme through CoI at low concentrations of ASC [23]; 5) continuous input of APX into the system at a non-constant rate, directly proportional to the difference between the initial enzyme concentration and the concentration of the active forms in each instant (see Eqn. (30)); the aim of this step was to obtain a stable level of APX activity under nonstress conditions. The APX rate equation is defined by the two steps of ASC oxidation to MDA, and then:

21 $$ {V}_{APX}={k}_2^{APX}\left[CoI\right]\left[ASC\right]+{k}_3^{APX}\left[ CoII\right]\left[ASC\right] $$

Other nonenzymatic reactions that participate in the pathway under study and which were included in our model are indicated in Fig. 1c (Eqs. (1)-(6)) [5, 8]. Briefly, spontaneous dismutation of MDA is necessary to supply DHA to DHAR to thus close the enzymatic cycle between APX and DHAR. Superoxide radicals spontaneously dismutate to O_2_ and H_2_O_2_ quite rapidly. DHA and superoxide are the most relevant molecules to contribute to the uncatalyzed GSSG production in vivo [24]. The spontaneous oxidation of ASC by H_2_O_2_ and O_2_^· −^ is also relevant. There are some reactions that proceed both spontaneously and enzymatically-driven, and therefore compete for the same substrates (SOD with Eq. (2), DHAR with Eq. (3), APX with Eq. (5)); in these cases, we numerically checked that the enzymatic reaction rate was higher than the rate of the corresponding chemical reaction under nonstress conditions. The rate constants corresponding to these reactions were considered apparent bimolecular rate constants. Most have been taken from the literature and are the same as those previously described (Table 1). However, the values for the macroscopic kinetic constants corresponding to a set of processes (i.e. *min* and *max* values for *a*_*i*_, *k*_11_, *k*_12_, *k*_13_, *k*_*N*,*cte*_, *k*_5_^*APX*^) were adapted to the present model in order to obtain reasonable results. The *k*_*3*_-value published [25], led to reaction (3) rates much higher than *V*_*DHAR*_, which considerably weakened the role of this enzyme in the pathway. For this reason, its value was adapted in the model. This possibility has been previously suggested [8] after considering that the rate constant for the reaction (3) in tissues would very likely be significantly lower than that determined in vitro in aqueous media.

**Table 1:** Relation of the kinetic parameters used to run the present model under nonstress conditions

Kinetic Parameter	Value	Unit	Reference
a_i_ (min)	3.5 x 10^6^	μM h^−1^	-
a_i_ (max)	4.5 x 10^6^	μM h^−1^	-
b	11	h	-
c	3π/2		-
∆	0.25	h	-
*k*_*cat*_^*GR*^	2,142,000	h^−1^	[5, 8]
*k*_*cat*_^*DHAR*^	511,200	h^−1^	[5, 8]
*k*_*cat*_^*MDAR*^	1,080,000	h^−1^	[20]
*k* ^*SOD*^	7.2 x 10^5^	μM^−1^ h^−1^	[5]
*k*_1_^*APX*^	43,200	μM^−1^ h^−1^	[2, 5]
*k*_2_^*APX*^	1.8 x 10^5^	μM^−1^ h^−1^	[2, 5]
*k*_3_^*APX*^	7,560	μM^−1^ h^−1^	[2, 5]
*k*_4_^*APX*^	2,520	μM^−1^ h^−1^	[2, 5]
*k*_5_^*APX*^	1	h^−1^	-
*K*_*m*,*GR*_^*NADPH*^	3	μM	[5, 8]
*K*_*m*,*GR*_^*GSSG*^	200	μM	[5, 8]
*K*_*i*_^*DHA*^ *K*_*m*,*DHAR*_^*GSH*1^	500	μM^2^	-
*K*_*m*,*DHAR*_^*DHA*^	70	μM	[5, 8]
*K*_*m*,*DHAR*_^*GSH*1^ + *K*_*m*,*DHAR*_^*GSH*2^	2,500	μM	[5, 8]
*K*_*m*,*MDAR*_^*MDA*^	1.4	μM	[20]
*K*_*m*,*MDAR*_^*NADPH*^	23	μM	[20]
*k*_*N*,*cte*_	5 x 10^−3^	μM^−1^	-
*k*_*11*_	30,000	h^−1^	-
*k*_*12*_	2,200	μM^−1^ h^−1^	-
*k*_*13*_	15,000	μM^−1^ h^−1^	-
*k*_*1*_	1,800	μM^−1^ h^−1^	[5, 48]
*k*_*2*_	720	μM^−1^ h^−1^	[5, 49]
*k*_*3*_	0.01	μM^−1^ h^−1^	-
*k*_*4*_	2,520	μM^−1^ h^−1^	[5, 8, 22]
*k*_*5*_	7.2x10^−3^	μM^−1^ h^−1^	[5, 8, 50]
*k*_*6*_	720	μM^−1^ h^−1^	[5, 8, 22]

Based on all these considerations, the complete system of ODEs corresponding to the model shown in Fig. 1 is the following:

22 $$ \frac{d\left[ NADPH\right]}{dt}=-{V}_{GR}-{k}_N\left[ NADPH\right]+0.5{F}_{12}-{V}_{MDAR} $$

23 $$ \frac{d\left[NAD{P}^{+}\right]}{dt}={V}_{GR}+{k}_N\left[ NADPH\right]-0.5{F}_{12}+{V}_{MDAR} $$

24 $$ \frac{d\left[GSH\right]}{dt}=2\cdot \left({V}_{GR}-{V}_{DHAR}-{k}_3\left[DHA\right]\left[GSH\right]-{k}_4\left[{O}_2^{\cdot -}\right]\left[GSH\right]\right) $$

25 $$ \frac{d\left[ GSSG\right]}{dt}=-{V}_{GR}+{V}_{DHAR}+{k}_3\left[DHA\right]\left[GSH\right]+{k}_4\left[{O}_2^{\cdotp -}\right]\left[GSH\right] $$

26 $$ \begin{array}{c}\frac{d\left[ASC\right]}{dt}={V}_{DHAR}+{k}_1{\left[MDA\right]}^2+{k}_3\left[DHA\right]\left[GSH\right]+{F}_{13}-{k}_2^{APX}\left[ASC\right]\left[CoI\right]-\\ {}-{k}_3^{APX}\left[ASC\right]\left[ CoII\right]-2{k}_5\left[{H}_2{O}_2\right]\left[ASC\right]-{k}_6\left[{O}_2^{\cdot -}\right]\left[ASC\right]+2{V}_{MDAR}\end{array} $$

27 $$ \frac{d\left[DHA\right]}{dt}=-{V}_{DHAR}+{k}_1{\left[MDA\right]}^2-{k}_3\left[DHA\right]\left[GSH\right] $$

28 $$ \begin{array}{c}\frac{d\left[MDA\right]}{dt}={k}_2^{APX}\left[ASC\right]\left[CoI\right]+{k}_3^{APX}\left[ASC\right]\left[ CoII\right]-2{k}_1{\left[MDA\right]}^2+\\ {}+2{k}_5\left[{H}_2{O}_2\right]\left[ASC\right]+{k}_6\left[{O}_2^{\cdot -}\right]\left[ASC\right]-{F}_{13}-2{V}_{MDAR}\end{array} $$

29 $$ \begin{array}{c}\frac{d\left[{H}_2{O}_2\right]}{dt}={V}_{SOD}-{k}_1^{APX}\left[{H}_2{O}_2\right]\left[APX\right]-{k}_4^{APX}\left[{H}_2{O}_2\right]\left[CoI\right]+{k}_2{\left[{O}_2^{\cdot -}\right]}^2+\\ {}+{k}_4\left[{O}_2^{\cdot -}\right]\left[GSH\right]-{k}_5\left[{H}_2{O}_2\right]\left[ASC\right]+{k}_6\left[{O}_2^{\cdot -}\right]\left[ASC\right]\end{array} $$

30 $$ \begin{array}{c}\frac{d\left[APX\right]}{dt}=-{k}_1^{APX}\left[{H}_2{O}_2\right]\left[APX\right]+{k}_3^{APX}\left[ASC\right]\left[ CoII\right]+\\ {}+{k}_5^{APX}\left({\left[APX\right]}_0-\left[APX\right]-\left[CoI\right]-\left[ CoII\right]\right)\end{array} $$

31 $$ \frac{d\left[CoI\right]}{dt}={k}_1^{APX}\left[{H}_2{O}_2\right]\left[APX\right]-{k}_2^{APX}\left[ASC\right]\left[CoI\right]-{k}_4^{APX}\left[{H}_2{O}_2\right]\left[CoI\right] $$

32 $$ \frac{d\left[ CoII\right]}{dt}={k}_2^{APX}\left[ASC\right]\left[CoI\right]-{k}_3^{APX}\left[ASC\right]\left[ CoII\right] $$

33 $$ \frac{d\left[AP{X}_i\right]}{dt}={k}_4^{APX}\left[{H}_2{O}_2\right]\left[CoI\right] $$

34 $$ \frac{d\left[{O}_2^{\cdot -}\right]}{dt}=-2{V}_{SOD}+{F}_{11}-2{k}_2{\left[{O}_2^{\cdot -}\right]}^2-{k}_4\left[{O}_2^{\cdot -}\right]\left[GSH\right]-{k}_6\left[{O}_2^{\cdot -}\right]\left[ASC\right] $$

## Methods

Simulated progress curves were obtained by numerical solutions of this nonlinear set of ODEs with the initial conditions indicated in Table 2. Numerical integration was performed with the help of the COPASI 4.7 software (Build 34) [14] using a deterministic algorithm (LSODA) that is able to deal with stiff ODEs [26]. COPASI is one of the most popular software applications for the simulation and analysis of biochemical networks. The resulting ODE model (15 days) consists in 13 species and 67 global quantities (kinetic parameters, enzymatic rates and fluxes).

**Table 2:** The initial conditions used to run the present model

Metabolite	Value	Unit	Reference
[DHAR]_0_	1.7	μM	[5, 8]
[MDAR]_0_	2	μM	[20]
[GR]_0_	1.4	μM	[5]
[SOD]_0_	50	μM	[5, 8]
[APX]_0_	40	μM	[2, 8]
[NADPH]_0_	110	μM	[5]
[NADP^+^]_0_	40	μM	[5]
[GSH]_0_	4,000	μM	[5]
[ASC]_0_	10,000	μM	[5, 8]

## Results and discussion

### Model performance under nonstress conditions

**Fig. 2:**
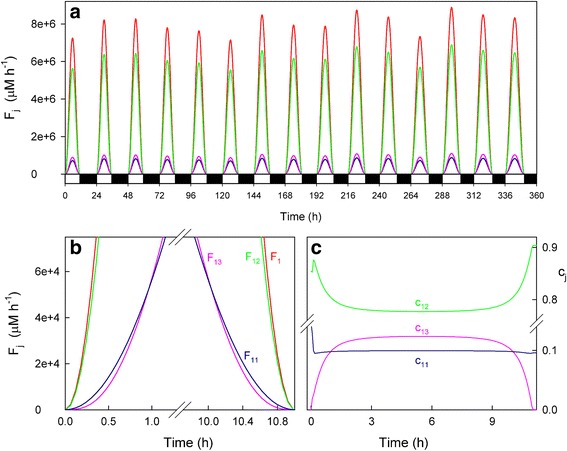
Simulated progress curves corresponding to the electron fluxes *F*_*j*_ (*j* = 1,11,12,13) and distribution coefficients *c*_*j*_ (*j* = 11,12,13). **a** Electron fluxes obtained in a run for 15 days using *b* = 11 h and ∆ = 0.25 h. Line colors are as indicated in panel **b**. In abscissa, white bars indicate light, while black indicate the dark. **b** Amplification of the data from Fig. 2a at dawn and dusk for the first day (the same every day). **c** Progress curves corresponding to the distribution coefficients *c*_*j*_ (*j* = 11,12,13) for the first day (the same every day). The values of the rate constants and initial conditions used are those indicated in Tables 1 and 2, except [MDAR]_0_ = 0

**Fig. 3:**
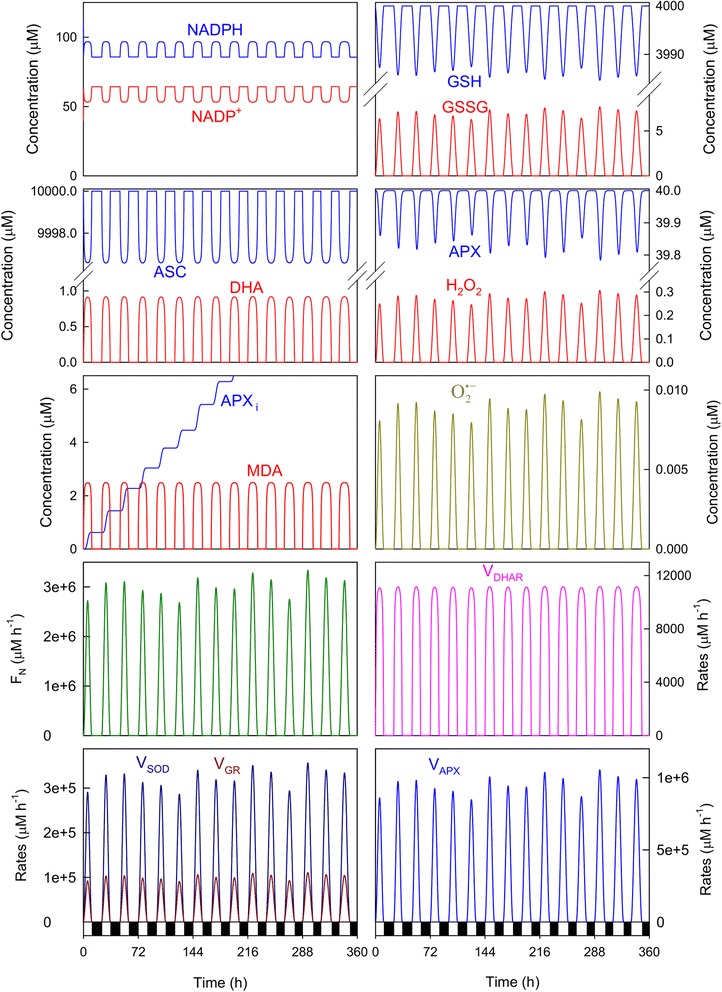
Simulated progress curves under non-stress conditions. Parametric conditions as indicated in Fig. 2. In abscissa, white bars indicate light, while black depict the dark

Light-dependent reactions in chloroplasts use solar power to reduce NADP^+^ to NADPH, which is necessary for CO_2_ fixation. Photoreduction of molecular oxygen to O_2_^· −^ and MDA to ASC also occurs competitively. Then the detoxifying efficiency of the ASC-GSH pathway can be evaluated by the relative distribution of the electron flux *F*_*1*_ among the three competitive processes (*F*_*11*_*, F*_*12*_ and *F*_*13*_). Figure 2 shows the results obtained herein for *F*_*j*_ (*j* = 1,11,12,13) (Fig. 2a and b) and *c*_*j*_ (*j* = 11,12,13) (Fig. 2c) by computational simulation under nonstress conditions while running the model for 15 consecutive days, by beginning with a photoperiod of 11 h (*b*) and increasing this latter (Δ) by 0.25 h/day (which would correspond approximately to a time period from February to June in Spain [13]). The *a*_*i*_ parameters were randomly obtained from Eq. (13) within the 3.5 x 10^6^ and 4.5 x 10^6^ μM h^−1^ range (which is equivalent to a maximum value of *F*_*1*_ in the range ~1.9-2.5 mM s^−1^ [8]), so that each run of the model is different. Under these conditions, the distribution of the electron flow among the three routes throughout the photoperiod was not constant (Fig. 2b and c), and the photochemical reduction of NADP^+^ (*F*_*12*_) was predominant despite a relatively low value of *k*_*12*_ was chosen (Table 1). Therefore, NADPH was synthesized in the model upon electron transport activation at sunrise, and the NADPH/NADP^+^ ratio was sufficiently high under the light conditions (see also Fig. 3). Thus it is observed that *c*_*12*_ was the most significant distribution coefficient throughout the photoperiod (*c*_*11*_ = ~10 %, *c*_*12*_ = ~78 %, *c*_*13*_ = ~12 % at noon) (Fig. 2c). The O_2_^· −^ photogeneration rate (*F*_*11*_) was only slightly higher than *F*_*13*_ at dawn and dusk (Fig. 2b), while during the remaining photoperiod, the photoreduction of ASC was higher than *F*_*11*_ to maintain ascorbate homeostasis. This is in agreement with the experimental observations made in leaves and chloroplasts under normal physiological conditions [27]. Under these parametric conditions, the maxima superoxide production rates were in the range 7.1 x 10^5^-8.8 x 10^5^ μM h^−1^ (~200-250 μM s^−1^), which are in the same order of magnitude as data previously reported [2, 8, 22].

Figure 3 shows the simulated progress curves that correspond to the chemical species and enzymatic activities involved in the model shown in Fig. 1 under the same parametric conditions as in Fig. 2. As expected according to the fact that midday electron fluxes were higher (in agreement with higher light intensities and temperatures), the concentrations and enzymatic activities involved in the ASC-GSH cycle peaked at noon, and were higher (or lower for NADP^+^, GSH, ASC and APX) than those in the early morning or near sunset. Two kinds of dynamic behavior can be observed from these plots. First, there were some chemical species like NADPH and NADP^+^, ASC, DHA and MDA, and enzymatic activities like *V*_*DHAR*_, whose levels were similar from day to day, regardless of solar irradiance intensity. Second, there was another group of chemical species and enzymatic rates, among which GSH and GSSG, H_2_O_2_, O_2_^· −^, APX, the Calvin-Benson cycle rate (*F*_*N*_), *V*_*SOD*_, *V*_*GR*_ and *V*_*APX*_ were included, whose levels were more closely related to *F*_*1*_ since a parallel variation was obtained. It is worth noting here that *V*_*GR*_ and *V*_*DHAR*_ should be equal in a stationary situation as a substrate cycle is established between them [6, 7]. However, the presence of the chemical steps in the model (Fig. 1c) led to a different situation, which was checked by simulations when these steps were absent (data not shown).

The numerical results indicated that ASC and GSH concentrations are maxima at night when ROS generation lowered. In contrast, the highest NADPH concentration was achieved during light conditions because its photoproduction from NADP^+^ predominated over its consumption for H_2_O_2_-detoxification (*F*_*12*_ *> F*_*11,*_ see Fig. 2a and b). The H_2_O_2_ concentration was low early in the morning and peaked at noon before lowering again throughout the afternoon and evening. This situation agrees with the experimental data measured in mangrove leaves [10] and tobacco leaves [28]. Greater APX inactivation was also obtained at noon as a consequence of the rise in the H_2_O_2_ levels (Fig. 3). At sunset, APX recovered through the *de novo* synthesis to reach to its original value under dark conditions. When a new photoperiod started, APX inactivation once again began. The amount of inactive APX (APX_i_) formed described a ladder-shaped plot where the greater *F*_*1*_ was, the more pronounced steps were. Under these parametric conditions, APX was the most active enzyme in the cycle.

### Model performance under high light conditions

**Fig. 4:**
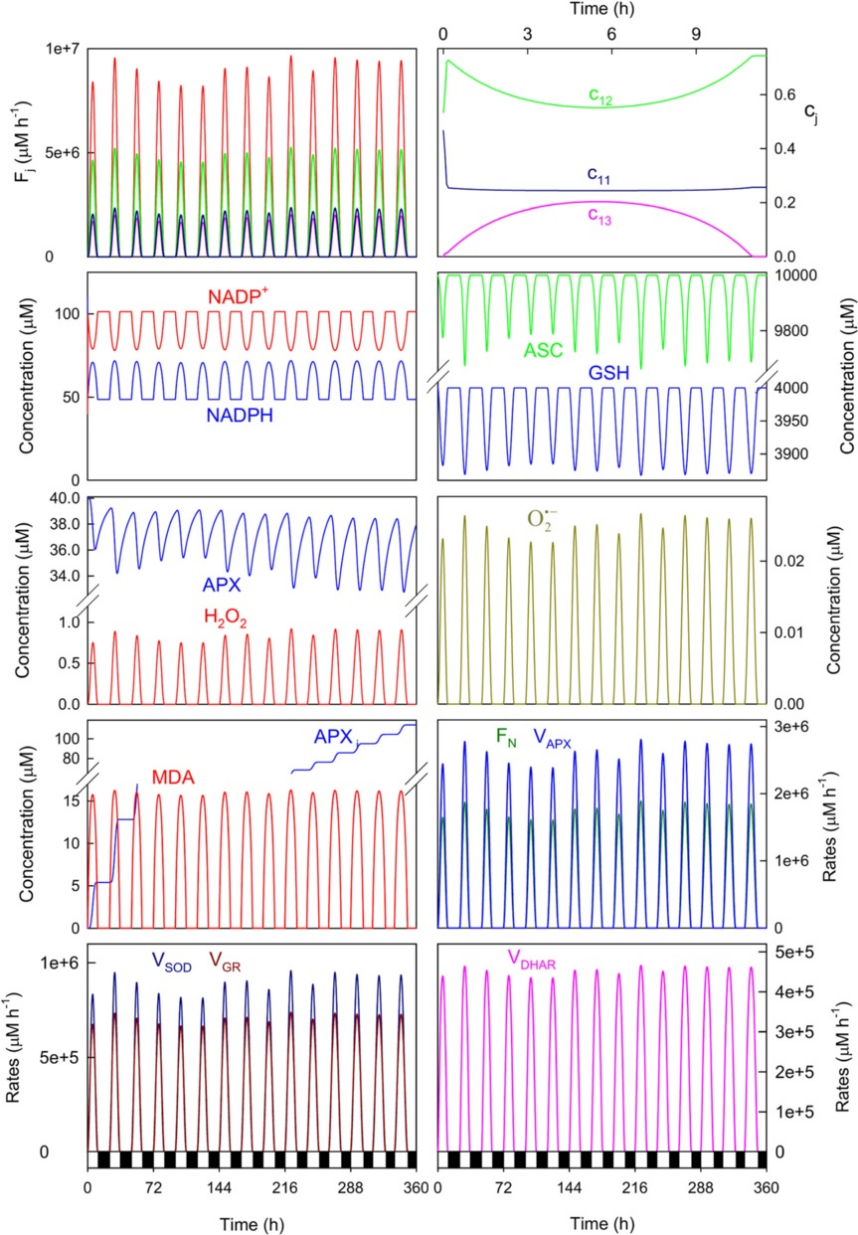
Simulated progress curves under the conditions chosen to simulate high-light conditions. The values of the rate constants and initial conditions used are those indicated in Tables 1 and 2, except *a*_*i*_ *(min)* = 4x10^6^ μM h^−1^, *a*_*i*_ *(max)* = 5x10^6^ μM h^−1^, *k*_*11*_ = 3.5x10^4^ h^−1^, *k*_*12*_ = 500 μM^−1^ h^−1^, *k*_*13*_ = 1,850 μM^−1^ h^−1^, *k*_5_^*APX*^= 0.1 h^−1^ and [MDAR]_0_ = 0. In abscissa, white bars indicate light, while black denote the dark. Line colors for *F*_*j*_ as indicated for *c*_*j*_. For greater clarity, *c*_*j*_ have been plotted only for the first day

In a continuously changing environment, plants find themselves under many different biotic and abiotic conditions from day to day, such as pathogen challenges, salinity, drought, temperature extremes, heavy metal toxicity, ozone, high-light intensity, etc., which can disrupt cell redox homeostasis and lead to substantial losses in crop yields and quality. Among these factors, light is an essential source of energy, but also a major source of abiotic stress which leads to ROS overgeneration [29] which, when combined with other stresses, is exacerbated [30]. Therefore, in order to study the dynamic behavior of our model under stress conditions, the *a*_*i*_ values were increased and randomly obtained within the range 4 x 10^6^ to 5 x 10^6^ μM h^−1^ from Eq. (13) (it is equivalent to maxima values of *F*_*1*_ in the range ~2.2-2.8 mM s^−1^). To increase the stress, the distribution of the electron flow among the three routes involved in the model was also changed so that a higher production of O_2_^· −^ was attained, along with greater APX photoinactivation, for which the rate constant corresponding to its *de novo* biosynthesis (*k*_5_^*APX*^) was also decreased (the values of the kinetic parameters which have been changed compared to those indicated in Table 1 have been included in the Fig. 4 legend). Figure 4 shows the results thus obtained for 15 consecutive days, beginning with a photoperiod of 11 h (*b*) with an increase of this latter of 0.25 h/day (as indicated in Fig. 2). The data obtained indicated a predominant electron flow for the photochemical reduction of NADP^+^ (*F*_*12*_), even though the *k*_*12*_-value was lowered to increase stress (*c*_*11*_ = ~24 %, *c*_*12*_ = ~55 %, *c*_*13*_ = ~21 % at noon). Under these parametric conditions, the maxima superoxide production rates were in the range 2.0 x 10^6^ – 2.4 x 10^6^ μM h^−1^ (~550-660 μM s^−1^), which are in the order of magnitude as data previously reported for stressed chloroplasts [8].

Distribution coefficients evolved differently to the previous parametric conditions. At *t* = 0, *F*_*1*_ was always split between *F*_*11*_ and *F*_*12*_, with *c*_*13*_ = 0 because it was assumed that [MDA]_0_ = 0 (Table 2). However under high light conditions, as *F*_*11*_ worked very early in the day to generate O_2_^· −^, *c*_12_ sharply increased to compensate *c*_*11*_, so *c*_*12*_ reached a maximum and *c*_*11*_ a minimum. Afterward, *c*_*13*_ began to increase because MDA was produced at the expense of ASC oxidation, so *c*_*12*_ decreased and *c*_*11*_ remained approximately constant. In the afternoon, the system recovered until the production of free radicals stopped at night. Under these conditions, *c*_*11*_ was always above *c*_*13*_.

Yet despite the system being subject to extremely stressful conditions, the NADPH levels were well buffered and regulated from day to day to peak at noon, although its concentration was lower than under the nonstress conditions (see Fig. 3). The differences between the maxima levels attained each day at noon were quite small. This result is interesting because it has been shown that chloroplastic NADPH concentrations change little after adjustment to light [31, 32], even in chloroplasts stressed with 10 μM paraquat [33], and also it is true that the NADPH levels are higher in the light than in the dark [34]. The system became more stressed as the consumption of antioxidants GSH and ASC was greater, with higher concentrations of GSSG, DHA, MDA, APX_i_, H_2_O_2_ and O_2_^· −^ attained at noon. The MDA levels achieved at noon remained approximately constant daily regardless of *a*_*i*_, which once again illustrates the system’s regulatory capacity. The enzymatic activities involved in the cycle were greater than under the previous conditions, which represents the defense mechanism of chloroplasts against increased ROS production. In fact, the overexpression of these enzymes has been shown to be linked to increased plant tolerance against different environmental stresses [35].

### Effects on the system of varying antioxidant enzyme activities

Although chloroplastic APX is the primary target of photooxidative damage [2], the activity of the other enzymes in the pathway could also be decreased, particularly in combination with other stressful environmental factors. Therefore, computer simulations were also run under the above high-light conditions by decreasing the catalytic constant of each enzyme to investigate the effect produced on the metabolites and fluxes of the metabolic network.

**Fig. 5:**
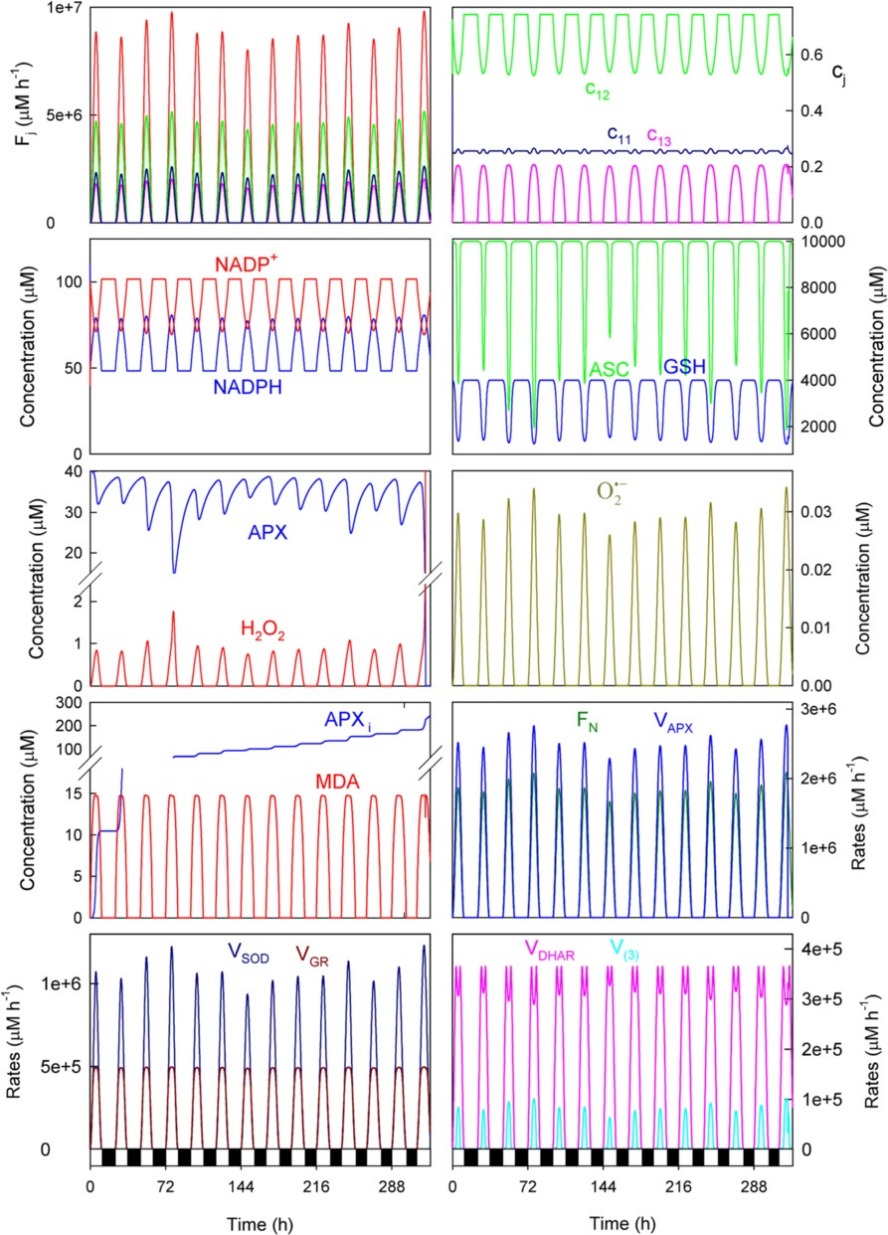
Simulated progress curves under high-light and GR-limiting conditions. The values of the rate constants and initial conditions were as indicated in Fig. 4, except *k*_*cat*_^*GR*^ = 420,000 h^−1^

Figure 5 shows the results obtained under GR-limiting conditions. The most marked effect was a substantial drop in the GSH/GSSG ratio at noon, which agrees with the physiological role of GR [24], and led to greater ASC consumption and higher H_2_O_2_ concentrations for the maximum solar irradiance hours (see Fig. 4). As a result, on day 14 (t = 320.4 h in the run shown herein), the H_2_O_2_ levels were well above a life-compatible level (in the order of mM), which gave rise to total APX inactivation and ASC depletion. The amount of formed APX_i_ was larger than that obtained under the previous conditions, and the O_2_^· −^ levels were higher (*c*_*11*_ shows an increase at noon under these stressful conditions), as was SOD activity. However less *V*_*DHAR*_ was observed, and even showed a minimum at noon as a result of lower GSH availability, which was accompanied by a subsequent increase in the noncatalyzed parallel reaction (3) rate (*V*_*(3)*_) to counteract this drop in *V*_*DHAR*_.

MDAR is another important enzyme in the H_2_O_2_-detoxifying pathway to help regenerate ASC from MDA at the expense of NAD(P)H. Therefore, and given the importance of ASC availability to keep APX active, the enzyme was included in the model at this point under these highly stressful parametric conditions. The results yielded by the computer after numerous runs indicated the vital role of ASC recycling in response to extremely hard environmental stress conditions, since the numerical data obtained (see Additional file 2) came much closer to those obtained under the nonstress conditions; e.g., the MDA maximum levels at noon lowered to 3–4.5 μM and antioxidants consumption was much lower. Furthermore, ASC recovery through MDAR also led to a slower electron flow for the photochemical MDA reduction (*F*_*13*_), along with a subsequent increase in *F*_*12*_ to regenerate the excess of NADPH consumed by MDAR.

**Fig. 6:**
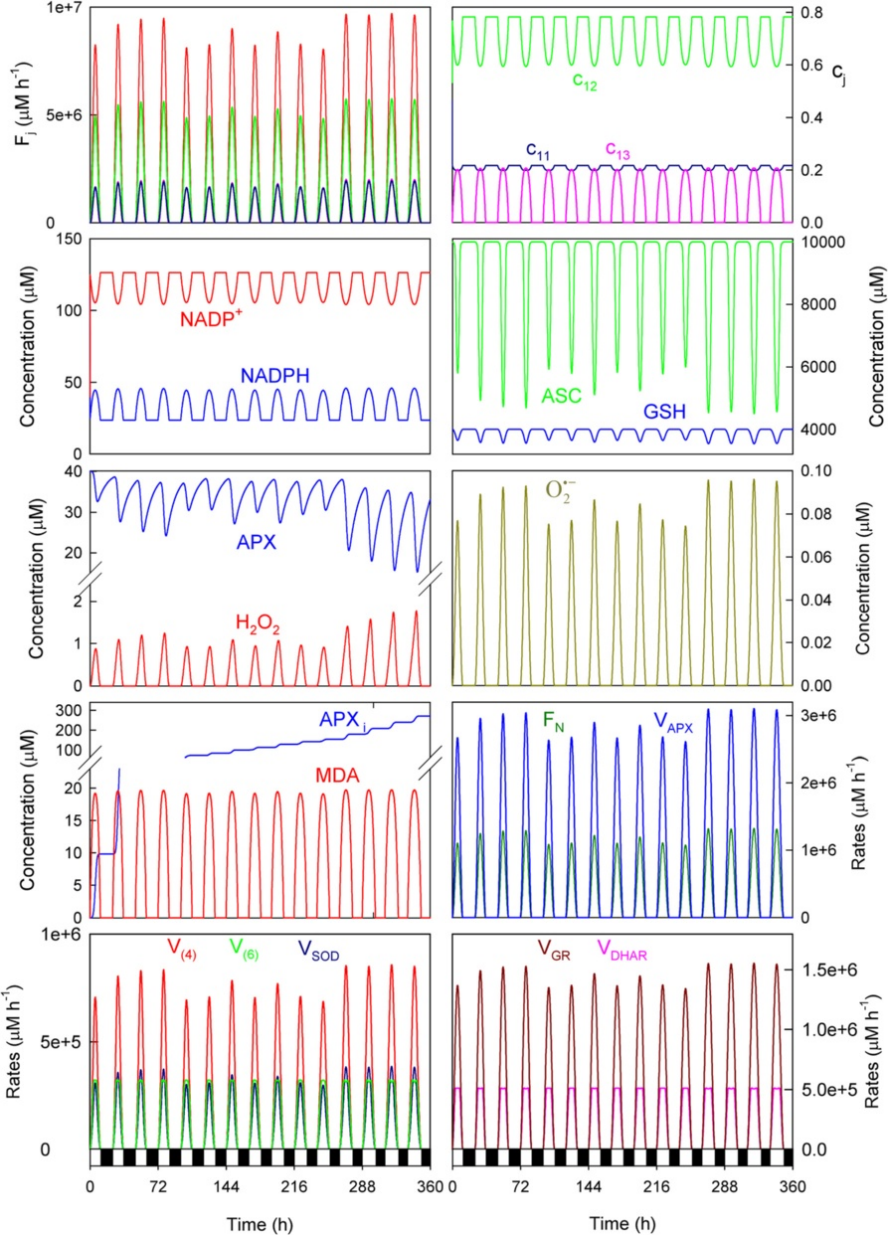
Simulated progress curves under high-light and SOD-limiting conditions. The values of the rate constants and initial conditions were as indicated in Fig. 4, except *k* ^*SOD*^ = 72,000 μM^−1^ h^−1^

When *k*^*SOD*^ was lowered in the system, the most meaningful effect was a considerable drop in the NADPH/NADP^+^ ratio (Fig. 6), higher MDA levels attained at noon and an increase in the rates of the uncatalyzed reactions (4) and (6) to remove the excess of O_2_^· −^ at the expense of GSH and ASC. The response of the system was to slow down the electron flow for superoxide photogeneration, with a minimum in *c*_*11*_ at noon (*c*_*11*_ = ~20 %, *c*_*12*_ = ~59.5 %, *c*_*13*_ = 20.5 % at noon). An increase in the rates of the other enzymes, DHAR, GR and APX was also observed, which also helped compensate the poorer catalytic activity of SOD.

**Fig. 7:**
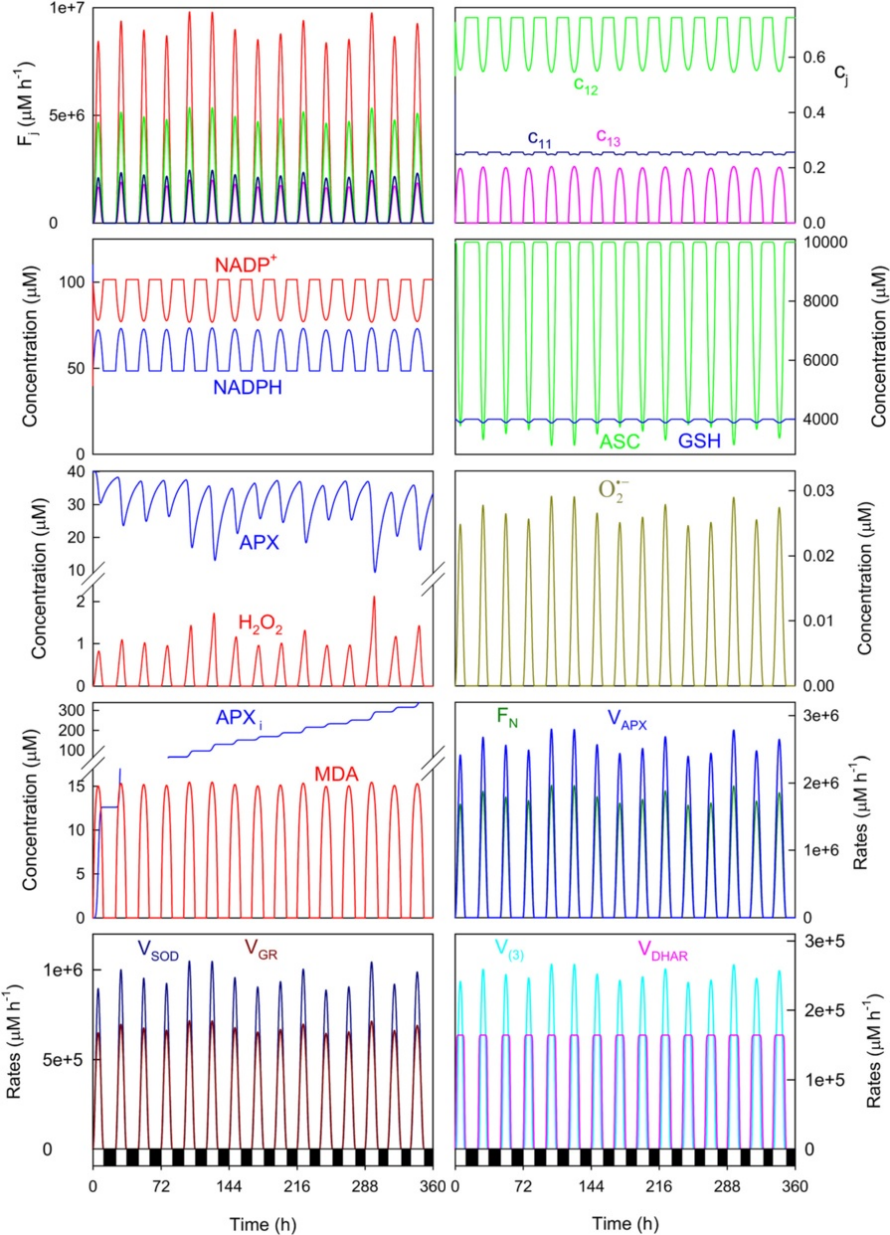
Simulated progress curves under high-light and DHAR-limiting conditions. The values of the rate constants and initial conditions were as indicated in Fig. 4, except *k*_*cat*_^*DHAR*^ = 160,000 h^−1^

A decrease in *k*_*cat*_^*DHAR*^ led to a greater consumption of antioxidants ASC and NADPH, although the GSH levels remained approximately constant (Fig. 7). As in the previous case, the rate of the spontaneous reaction (3), which is parallel to that catalyzed by DHAR, became higher. Increased ASC consumption led to greater APX inactivation at noon, while H_2_O_2_ and O_2_^· −^ levels were higher (see Fig. 4), although the system showed sufficient capacity to remove it.

For these two enzymes, SOD and DHAR, the catalytic constants could be decreased by several orders of magnitude without a significant amount of H_2_O_2_ being accumulated due to the counteracting effect of the spontaneous parallel steps.

### Model performance under NADP^+^-limiting conditions

In water or salt stress situations, a reduction in stomatal conductance occurs to limit water loss [36, 37]. Stomatal closure lowers the CO_2_ concentration available for the Calvin-Benson cycle, and consequently NADP^+^ availability to accept the electrons from PSI [2]. This means that more electrons from the electron transport chain in the chloroplast are used for O_2_ photoreduction, which leads to greater ROS generation (redox poising) [38, 39]. This was simulated in the model by decreasing the apparent rate constant for the Calvin-Benson cycle, *k*_*N,cte*_, and *k*_*cat*_^*GR*^, both under the standard conditions indicated in Table 1, and under the stressful high-light conditions (the parametric conditions indicated in Fig. 5), in the absence and presence of MDAR.

**Fig. 8:**
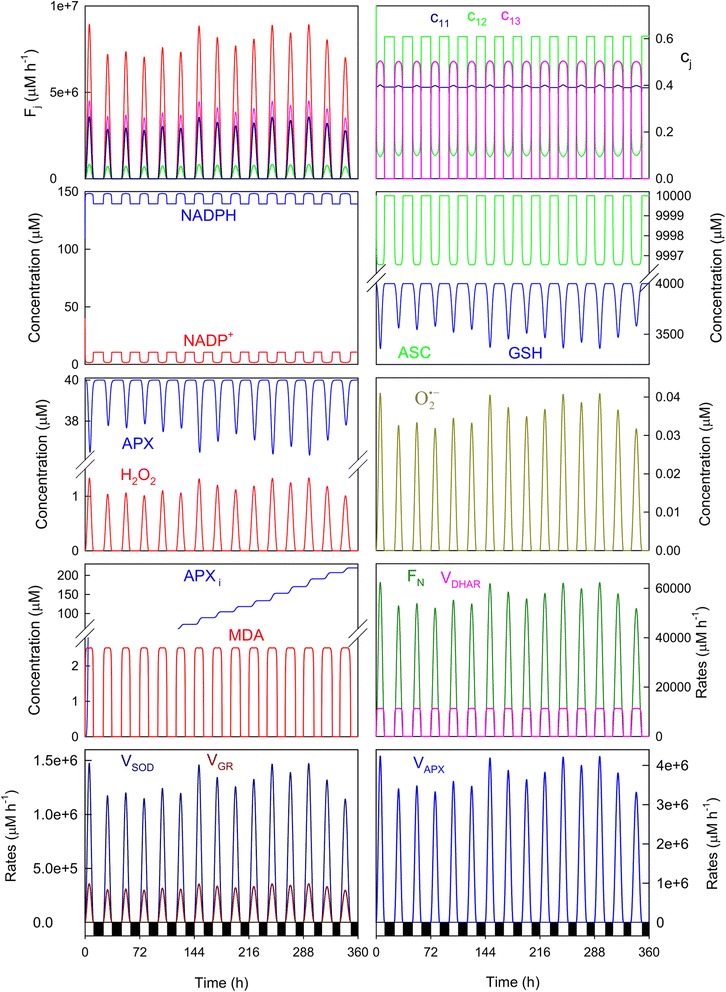
Simulated progress curves under NADP^+^-limiting conditions. The values of the rate constants and initial conditions used were those indicated in Tables 1 and 2, except *k*_*cat*_^*GR*^= 420,000 h^−1^, *k*_*N,cte*_ = 5.0x10^−4^ μM^−1^ and [MDAR]_0_ = 0

Under the standard conditions indicated in Table 1, and in the absence of MDAR, a change in the distribution of the electron flow from PSI was observed, which was in agreement to that previously indicated as *F*_*13*_ > *F*_*11*_ > *F*_*12*_ (Fig. 8). NADP^+^ levels were in fact considerably lower than those obtained under nonstress conditions (see Fig. 3). The most important differences with respect to the data obtained under nonstress conditions were higher GSH consumption, greater H_2_O_2_ and O_2_^· −^concentrations at noon, *F*_*N*_ considerably lowered, and *V*_*SOD*_ and *V*_*APX*_ were higher. However, MDA levels were in the same order of magnitude.

**Fig. 9:**
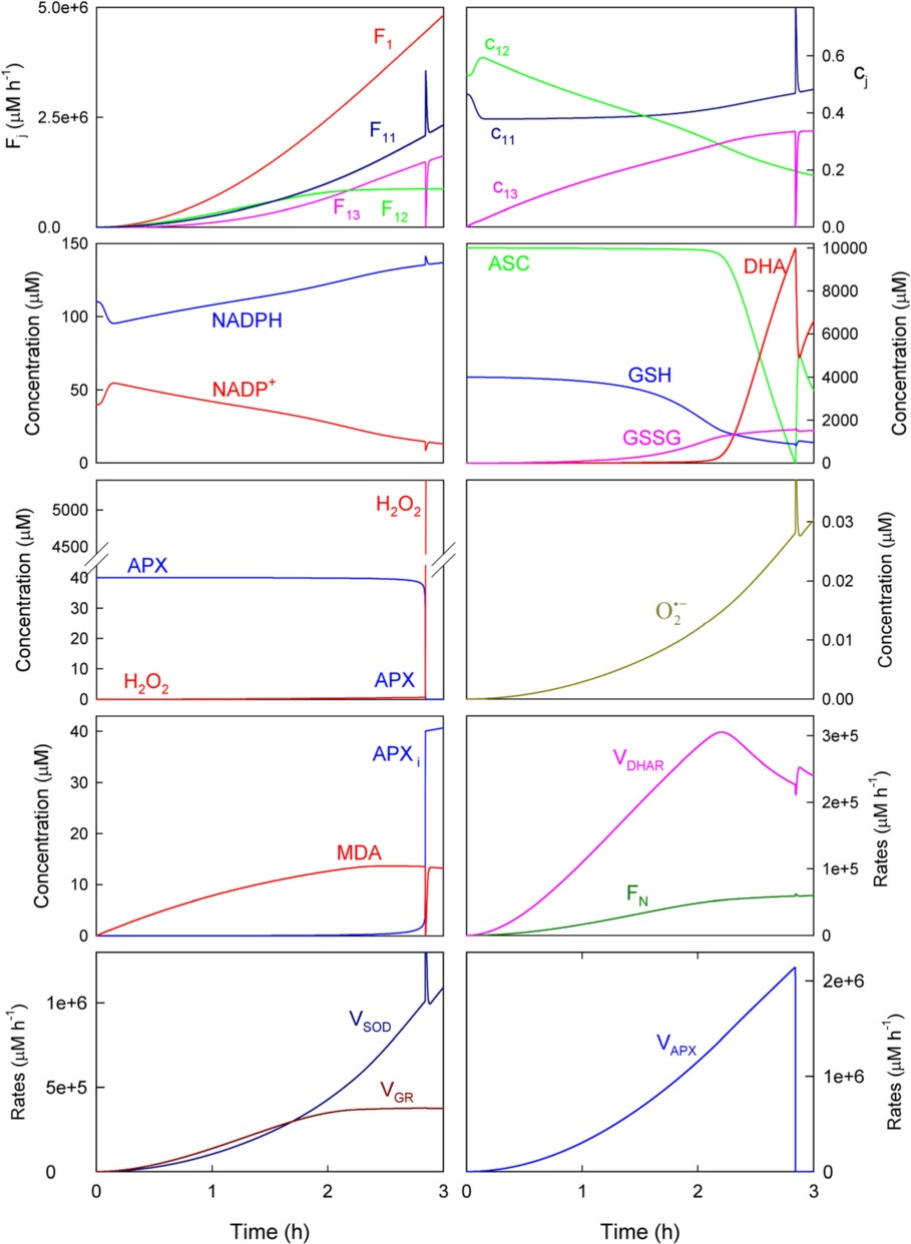
Simulated progress curves under high-light and NADP^+^-limiting conditions. The values of the rate constants and initial conditions used were those indicated in Fig. 5, except *k*_*N,cte*_ = 5.0x10^−4^ μM^−1^

Under high-light conditions and in the absence of MDAR, the distribution of the electron flow was in the order of *F*_*11*_ > *F*_*13*_ > *F*_*12*_ (i.e. the superoxide production rate was higher than the ASC and MDA photoreduction rates, except early in the morning) (Fig. 9), which resulted in extremely high H_2_O_2_ concentrations always being attained on the first day in each run. NADP^+^concentration increased early in the morning (in parallel with *c*_*12*_), but quickly decreased. There is an instant (t = 2.84 h in the run here shown) in which APX was totally inactivated and ASC was totally depleted (although it is possible to observe an increase in the ASC concentration after that, it has no biological sense. It is due to the spontaneous redox steps involved in the model; however, it must be taken into account that all of these recoveries observed after APX inactivation are produced at H_2_O_2_ concentrations above a life-compatible level). In this way, simulations comprehensibly revealed the importance of a tight regulation of the NADPH/NADP^+^ ratio to ensure sufficient NADP^+^ levels available to accept electrons in the electron transport chain [32].

**Fig. 10:**
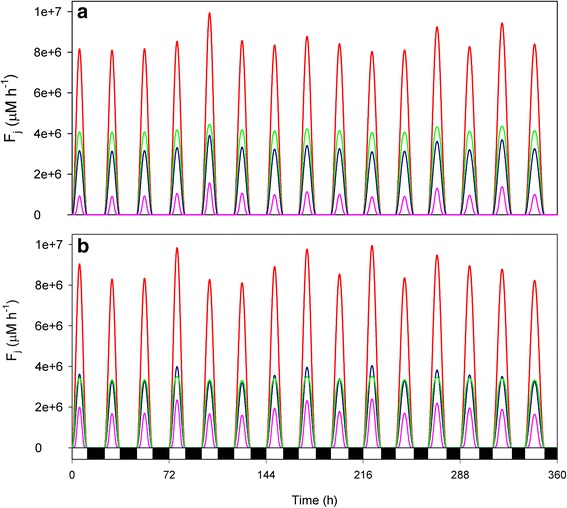
Electron flow distribution under high-light and NADP^+^-limiting conditions in the presence of MDAR. **a** [MDAR]_0_ = 2 μM, **b** [MDAR]_0_ = 1.3 μM. The rest of parameters were as indicated in Fig. 9. Other metabolite concentrations, fluxes and enzymatic rates are shown in Additional files 3 and 4, respectively, for conditions (**a**) and (**b**)

However, if MDAR was included under these fatal parametric conditions, the distribution of the electron flow changed to: *F*_*12*_ > *F*_*11*_ > *F*_*13*_ (Fig. 10a). Once again, a drop in *F*_*13*_ was observed at the expense of the increase in *F*_*12*_ as a result of ASC recycling through MDAR. This situation led to physiologically more adequate levels of H_2_O_2_, O_2_^· −^ and MDA at noon, and to a less significant consumption of GSH and ASC (although the system is still quite stressed, see Additional file 3). However, when this *in silico* experiment was repeated with a lower MDAR concentration, the distribution of the electron flow differed, and even differed from day to day at the time of maximum solar irradiance (Fig. 10b). In this scenario, the electron flow deviated mainly toward O_2_^· −^ generation (*F*_*11*_) every day, but at noon *F*_*12*_ > *F*_*11*_ was obtained for the days with lower solar irradiance (2, 3, 5, 6, 11, 15 in this run), whereas *F*_*11*_ > *F*_*12*_ for the days with greater solar irradiance (1, 4, 7, 8, 10, 12, 13, 14). ASC and GSH consumption was more meaningful (see Additional file 4), which led to higher levels of MDA, DHA and GSSG at noon.

All these results indicate that plants displaying increased activity in the Calvin-Benson cycle will better resist excessive light, i.e., the Calvin-Benson cycle, besides providing sugars, is also regenerating electron acceptors (NADP^+^ in the model) to thus represent chloroplast self-protection. In fact this is what actually happens. C4 and CAM plants are the best adapted to arid, hot, high-light environments because they possess greater photosynthetic efficiency than C3 plants [40]. It has also been proposed that one of the reasons for the successful adaptation of Antarctic vascular plants to high light/low temperature conditions is the robustness of their CO_2_ assimilation machinery [41]. This is also the role played by photorespiration in plants as a way to use the excess ATP and NADPH generated in thylakoids, and therefore to dissipate excess energy to prevent over-reduction of the photosynthetic electron chain and consequent damage in the photosynthetic apparatus [42]. As a matter of fact, it is well-known that the Calvin-Benson cycle enzymes subject to regulation by reduced thioredoxin are activated by reduction in the light and deactivated by oxidation in the dark. This mechanism has been demonstrated for several chloroplast enzymes, including fructose-1,6-bisphosphatase, sedoheptulose-1,7-bisphosphatase [43], phosphoribulokinase, NADP^+^-glyceraldehyde-3-phosphate dehydrogenase, Rubisco activase and ATPsynthase [44]. Evidently, this activation has a limit after which the electron transport chain becomes saturated and electrons are deflected toward dioxygen reduction.

By last, the mathematical model developed herein is a very useful tool to understand the elements that determine diurnal fluctuations in the chemical species involved in ROS generation-detoxification in chloroplasts. This study can be extended in different ways. One important aspect has been mentioned above, which would allow to make predictions of the adaptability of plants to different solar irradiance conditions. However, this would require knowledge of the model’s kinetic parameters per plant type. Another obvious aspect is to add new steps to the model to study the relation with other nearby metabolic pathways. The ASC-GSH pathway is closely related to the electron transport chain in the chloroplast, which is involved in ATP production for photosynthesis. Ongoing research looks at the relation between photosynthesis and the ASC-GSH pathway. Another very interesting aspect would be to consider the diffusion and compartmentalization processes of chemical species to model the asynchronous metabolite supplies to enzymes. Previously, we modeled the adenylate energy system and ATP production at the systemic cellular level with a system of delay-differential equations to take into account different time scales within the cell [45]. Finally, the present model can be extrapolated to other metabolic pathways to quantitatively analyze the effect of sunlight on their metabolism.

## Conclusions

In this paper, a mathematical model able to simulate not only the distribution of the electron flow from PSI in the chloroplast, but also the dynamics of the chemical and enzymatic reactions involved in the ASC-GSH pathway has been developed, which takes into account for the first time the succession of days and nights. The metabolic processes involved in the network have been described by a nonlinear system of ordinary differential equations in which the enzymatic rate equations and biochemical kinetic parameters have been retrieved from previously reported experimental data. One important novelty in the numerical results here shown is that metabolite concentrations and enzymatic activities do not evolve toward a steady-state level, but display oscillatory behavior, which is dependent on the time of day. The results here obtained clearly highlight the importance of the distribution of electron fluxes through the system for the detoxifying efficiency of the ASC-GSH pathway. The model can help to understand the elements that determine diurnal fluctuations in the chemical species involved in ROS generation-detoxification in chloroplasts. This approach can provide strategies to analyze plant defense mechanisms against oxidative stress and can be extrapolated to other metabolic pathways to quantitatively analyze the effect of sunlight on the metabolism.

### Availability of supporting data

The data sets supporting the results of this article are available in BioModels Database [46], with model identifier MODEL1503250002 [47].

## Additional files

Additional file 1:
**Fitting the average daily global clear-sky solar irradiance data to Eq. (** 11 **).** Data were taken from [13] after considering these parameters: geographic coordinates = 40° 25' 0'' North, 3° 42' 1'' West (Madrid, Spain), month = September, inclination of plane = 35° and orientation (azimuth) of plane = 0°. Dots represent the real solar irradiance data (adapted so that the photoperiod starts at time = 0) and the line corresponds to the nonlinear regression analysis. Data were fitted by the SigmaPlot Scientific Graphing Software for Windows, version 13.0 (2014, Systat Software, Inc.). (TIF 187 kb) Additional file 2:
**Simulated progress curves under high-light and GR-limiting conditions in the presence of 2 μM MDAR.** Other parametric conditions as indicated in Fig. 5. (TIF 1969 kb) Additional file 3:
**Simulated progress curves under NADP** ^**+**^ **-limiting and high-light conditions in the presence of 2 μM MDAR.** Parametric conditions as indicated in Fig. 10A. (TIF 1806 kb) Additional file 4:
**Simulated progress curves under NADP** ^**+**^ **-limiting and high-light conditions in the presence of 1.3 μM MDAR.** Parametric conditions as indicated in Fig. 10B. (TIF 1938 kb)

## Abbreviations

APX: ascorbate peroxidase
APX_i_: ascorbate peroxidase inactive
ASC: ascorbate
DHA: dehydroascorbate
DHAR: dehydroascorbate reductase
GR: glutathione reductase
GSH: reduced glutathione
GSSG: oxidized glutathione
MDA: monodehydroascorbate
MDAR: monodehydroascorbate reductase
ODEs: ordinary differential equations
PSI: photosystem I
PSII: photosystem II
ROS: reactive oxygen species
SOD: superoxide dismutase
