# Supplementary figures and images for: Modeling the ascorbate-glutathione cycle in chloroplasts under light/dark conditions
Source: BMC Syst Biol. 2016 Jan 22;10:11. doi: 10.1186/s12918-015-0239-y (PMC4722729; doi:10.1186/s12918-015-0239-y)

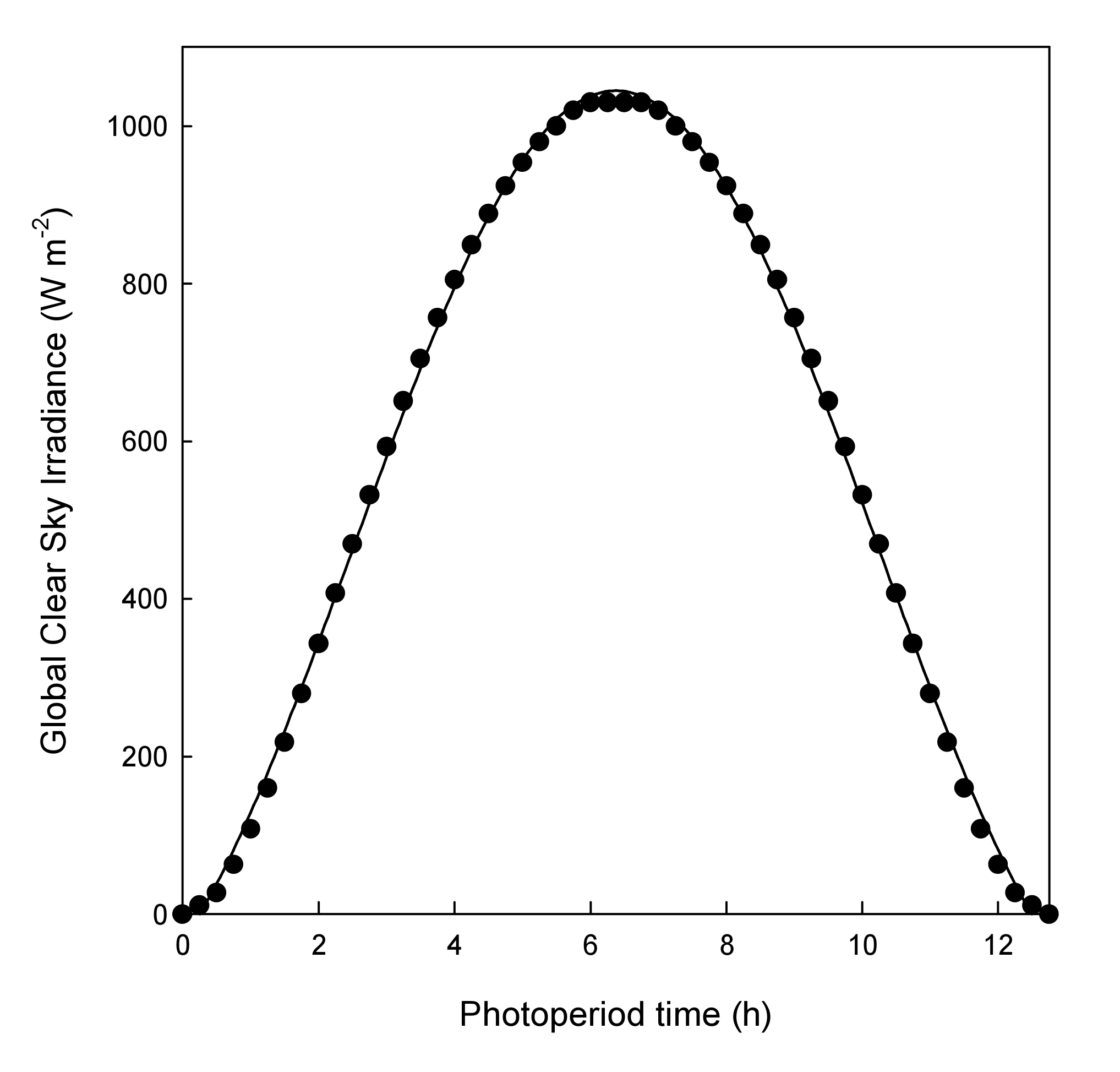

Supplement: Additional file 1: — Fitting the average daily global clear-sky solar irradiance data to Eq. ( 11 ). Data were taken from [13] after considering these parameters: geographic coordinates = 40° 25' 0'' North, 3° 42' 1'' West (Madrid, Spain), month = September, inclination of plane = 35° and orientation (azimuth) of plane = 0°. Dots represent the real solar irradiance data (adapted so that the photoperiod starts at time = 0) and the line corresponds to the nonlinear regression analysis. Data were fitted by the SigmaPlot Scientific Graphing Software for Windows, version 13.0 (2014, Systat Software, Inc.). (TIF 187 kb) [file 12918_2015_239_MOESM1_ESM.tif]

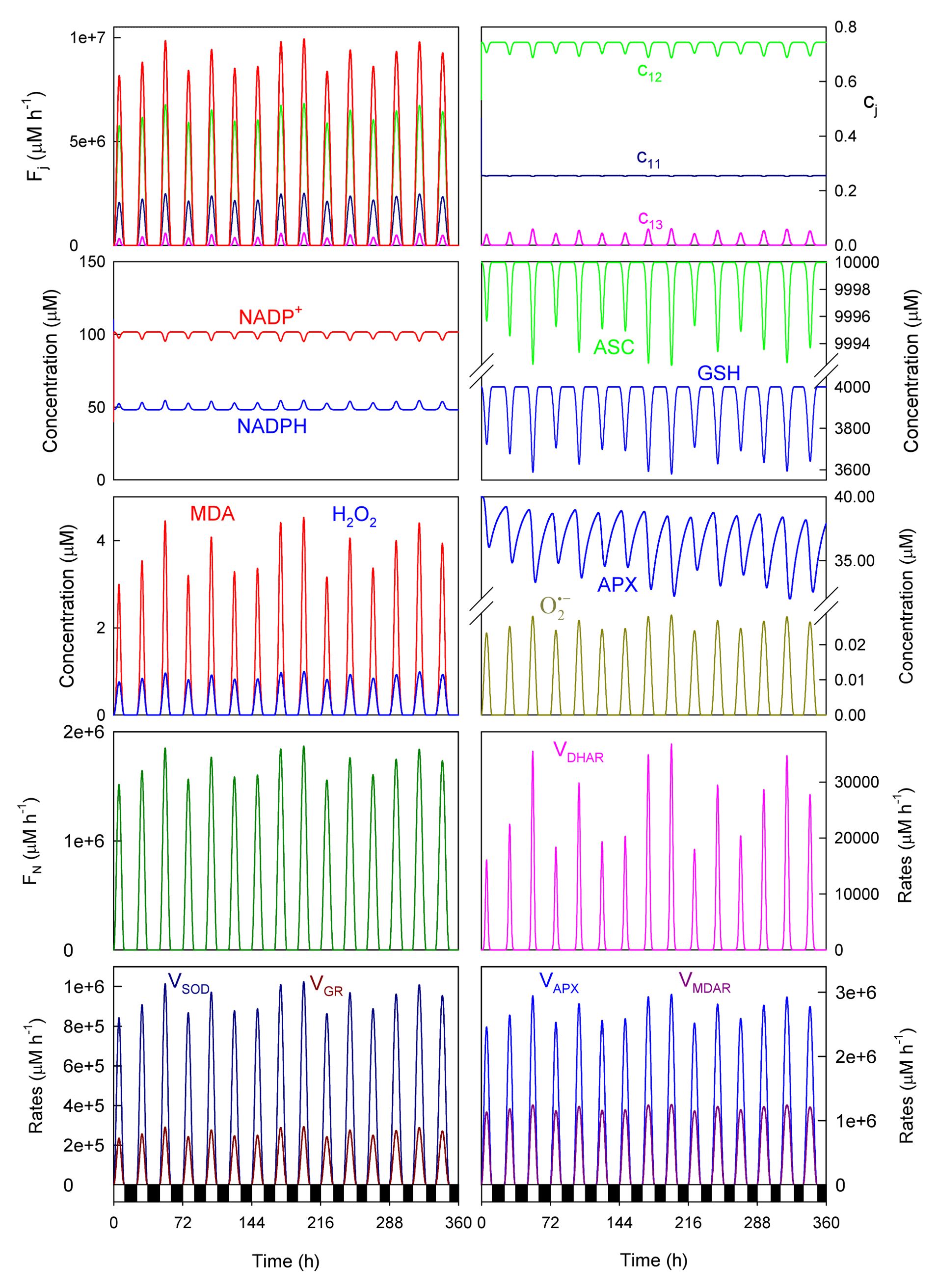

Supplement: Additional file 2: — Simulated progress curves under high-light and GR-limiting conditions in the presence of 2 μM MDAR. Other parametric conditions as indicated in Fig. 5. (TIF 1969 kb) [file 12918_2015_239_MOESM2_ESM.tif]

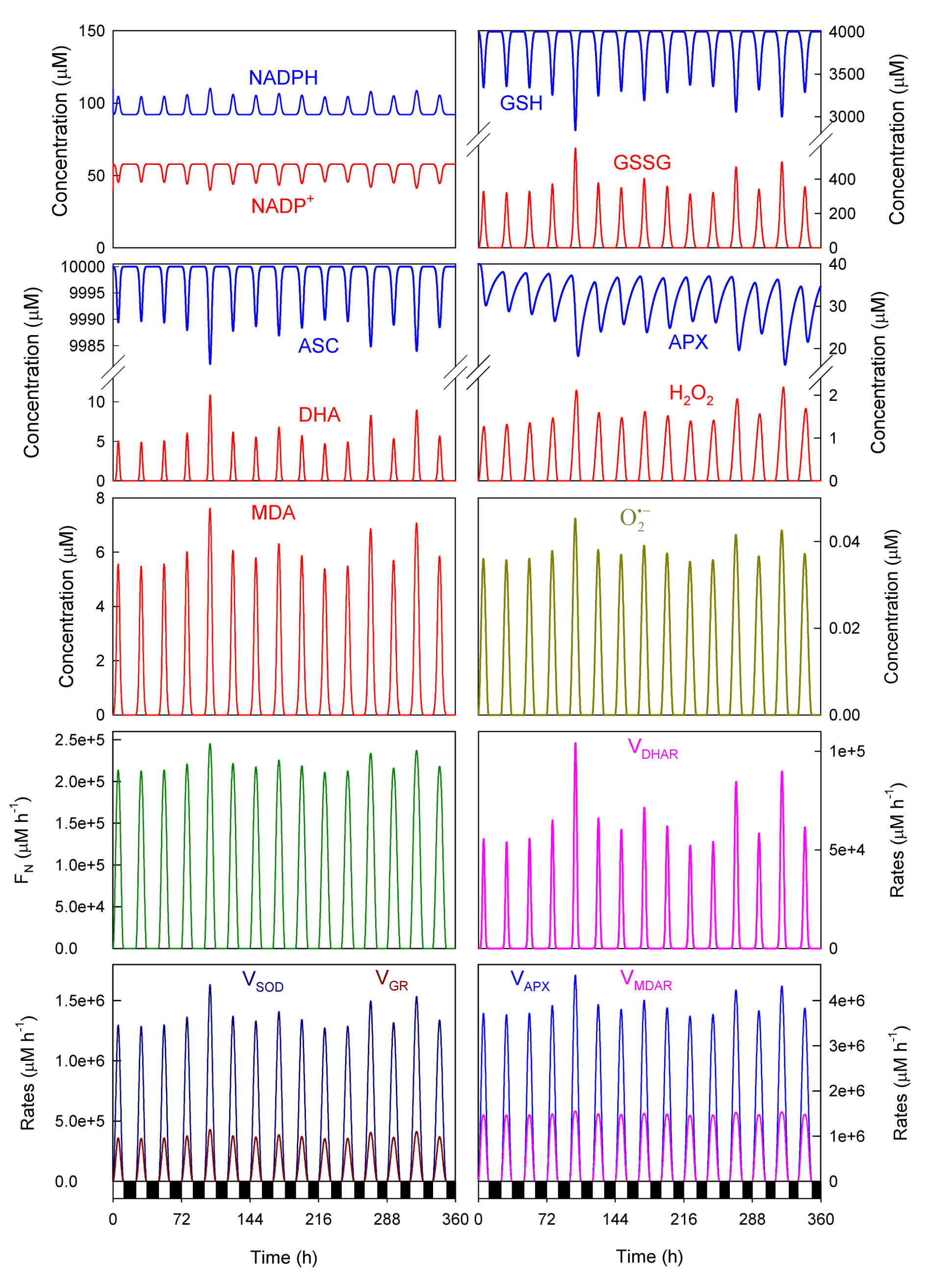

Supplement: Additional file 3: — Simulated progress curves under NADP + -limiting and high-light conditions in the presence of 2 μM MDAR. Parametric conditions as indicated in Fig. 10A. (TIF 1806 kb) [file 12918_2015_239_MOESM3_ESM.tif]

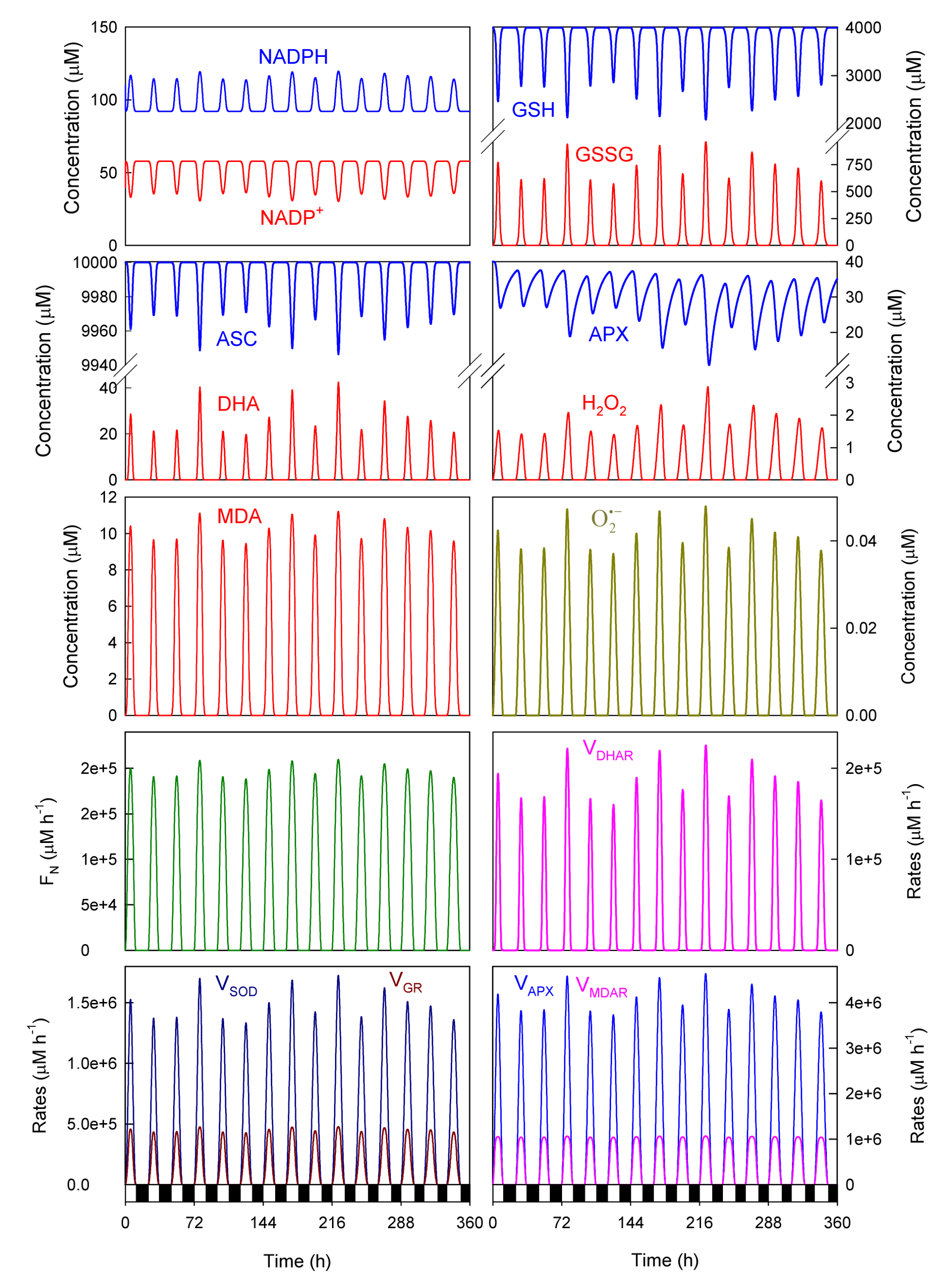

Supplement: Additional file 4: — Simulated progress curves under NADP + -limiting and high-light conditions in the presence of 1.3 μM MDAR. Parametric conditions as indicated in Fig. 10B. (TIF 1938 kb) [file 12918_2015_239_MOESM4_ESM.tif]
